# Maternal Responsive Parenting Trajectories From Birth to Age 3 and Children’s Self-Esteem at First Grade

**DOI:** 10.3389/fpsyg.2022.870669

**Published:** 2022-04-28

**Authors:** Yeon Ha Kim

**Affiliations:** Department of Child and Family Studies, Kyung Hee University, Seoul, South Korea

**Keywords:** responsive, trajectory, self-esteem, parenting, first grade

## Abstract

This paper examines the quality and stability of the responsive parenting practices of mothers with infants and the longitudinal links between these practices and children’s self-esteem. Using data presented by the Panel Study on Korean Children, this study identified Korean mothers’ responsive parenting trajectories from birth to age three and examined their associations with children’s self-esteem at first grade. Korean mothers developed one of three responsive parenting patterns from birth to age three: low (19.0%), moderate (66.0%), or high (15.0%). Children’s self-esteem differed according to their mother’s responsive parenting trajectory. First-graders with mothers displaying the low responsive parenting trajectory were more likely to have lower self-esteem than children of mothers with the moderate responsive parenting trajectory and children of mothers with the high responsive parenting trajectory. The longitudinal link between mother-reported responsive parenting patterns during infancy and child-reported self-esteem at first grade was verified. This finding highlights the significance of early responsive parenting from mothers as a predictor of the self-esteem of children in later developmental stages.

## Introduction

Self-esteem plays a key role in an individual’s psychological foundation and deeply influences major life outcomes (Orth et al., 2012). For that reason, identifying predictors of self-esteem has been an essential topic in psychology, education, and mental health research. Many studies have focused on the role of parents in developing children’s self-esteem. The general purpose of these studies was to portray the best parenting types or styles for fostering children’s positive self-esteem (Tam et al., 2012; Moghaddam et al., 2017). Thus far, the authoritative parenting style (i.e., high warmth and high control) and the permissive parenting style (i.e., high warmth and low control) have been positively linked to high self-esteem in children and adolescents (Milevsky et al., 2007; Pinquart and Gerke, 2019).

Considering that the common feature of both the authoritative and permissive styles is high warmth, the key to fostering positive self-esteem may be in providing affectionate, immediate and appropriate responses contingent on children’s needs. The term “responsiveness” has been used to describe responding with understanding and support to fulfill the needs and goals of a partner (Reis and Clark, 2013). The degree of responsiveness in parental behaviors is a key indicator of the quality of parenting (Knauer et al., 2019). High responsiveness in parenting has been known to strengthen parents’ relationships with their children and support children’s healthy development (Raval et al., 2001; Knauer et al., 2019). Meanwhile, it has been found that the quality, duration, and timing of experiences result in different human development outcomes (Linstead et al., 2017; Milner et al., 2018). More intense experiences over a longer duration at earlier stages of development result in more significant developmental differences. Who is associated with the experiences also matters. The profound impact of maternal parenting on child outcomes has been well validated (Milevsky et al., 2007; Wittig and Rodriguez, 2019). When we adapt these developmental notions to parenting and children’s self-esteem, differences in the intensity and stability of responsiveness in maternal parenting during infancy may yield differences in children’s self-esteem.

Among the many viewpoints on the causal mechanism between early responsive parenting and children’s self-esteem development, the most classic view may be the attachment perspective. In the attachment perspective, the quality of parenting contributes to the development of the internal working model concerned with interpreting the self and others (Bowlby, 1982). If parents have been highly sensitive to and available for the child’s needs, the child constructs a model of the self as worthy and lovable. Conversely, if parents have not been sensitive and accessible, the child interprets the self as unworthy and unacceptable (Bretherton et al., 1990). The links between attachment and self-esteem in adolescents and adult populations has been verified (Laible et al., 2004; Curran et al., 2021). For example, Laible et al. (2004) reported that a direct path exists between secure attachment and high self-esteem among college students in the United States. Curran et al. (2021) investigated how attachment styles were related to self-esteem in mother–adult child dyads in the United States. They reported that the secure attachment style was linked to high self-esteem in both generations.

The contemporary claim is that early responsive parenting builds a sound brain foundation for supporting children’s healthy social, emotional, and cognitive development (Belsky and De Haan, 2011). Bernier et al. (2016) investigated the links between the observed quality of mother–infant interactions and brain development in a normative sample of 352 mother–infant dyads. They reported that higher-quality mother–infant interactions predicted a higher frontal resting electroencephalography (EEG) power in infants, indicating more neural activity. Chen et al. (2021) investigated the neural bases of self-esteem in school-aged children. They found that children’s self-esteem was positively related to spontaneous activity in the right dorsolateral prefrontal cortex (dlPFC). They suggested that dlPFC might be a core brain region involved in promoting self-related cognitive processing in high self-esteem children. Considering the claim that early intensive and positive experiences during infancy can alter the brain’s functions and structures (Als et al., 2004), it is likely that high-quality responsive parenting from mothers during infancy enhances children’s brain structure related to self-esteem. Moreover, the impact embedded in the neurological foundation will extend into later developmental periods and will be observed as forming positive self-esteem.

Meanwhile, to clearly understand the role of maternal responsive parenting on children’s self-esteem development, identifying distinct sub-groups of mothers sharing a similar stability and intensity pattern of responsiveness can be effective (Ram and Grimm, 2009). Some studies, taking a person-centered perspective, have identified latent profiles of parenting behaviors among mothers with young children (Cook et al., 2012; Paschall and Mastergeorge, 2018; Farkas et al., 2020). For example, Cook et al. (2012) identified three latent groups with distinct parenting practices among Early Head Start mothers across three points in time when children were 14, 24, and 36 months: developmentally supportive, unsupportive, and negative. They reported that mothers’ latent group memberships were stable across these three points. Farkas et al. (2020) identified three different clusters among 90 Chilean mothers sharing similar parenting competencies at two different points in time, when children were at 12 and 30 months: highly competent, average competent, and poorly competent. Unlike the claims of Cook et al. (2012), the Chilean mothers increased their parenting competences. Only 16.7% of the mothers classified into the poorly competent group when children were at 12 months remained in the same group when children were at 30 months. The other mothers moved into the average or highly competent groups. The results of these studies imply that the quality of parenting behaviors cannot be explained with a single trend or profile and that there are some groups of mothers who deviate from normative trends in the quality and stability of their parenting behaviors.

However, few studies have endeavored to capture both within-group changes and between-group differences in responsive parenting among a normative sample of mothers. Previous studies in general have tried to identify a single trajectory representing the parenting practices of at-risk or clinical populations (Bornstein et al., 2008; Kim et al., 2010; Ettinger et al., 2018) or have searched for course modifiers (Azak and Raeder, 2013). Like other behaviors or psychological traits such as depressive symptoms (Kim, 2017) or alcohol use (De Genna et al., 2017), mothers’ parenting practices can vary in stability and intensity. Mothers with infants may be classified into multiple groups, each sharing similar stability and intensity of responsiveness. Group differences in the stability and intensity of mothers’ parenting practices indicates that the children of mothers in each group experience distinct parenting practices. Considering that the stability and intensity of experiences is associated with developmental outcomes (Liu et al., 2021), distinct trajectories of maternal responsive parenting can be a source of differences in children’s self-esteem in later years.

To sum up, the present study will investigate the associations between mothers’ early responsive parenting trajectories and their children’s self-esteem at first grade using a normative nationwide Korean sample. Self-esteem emerges in an individual’s early years and changes across one’s lifespan according to important life events or transitions (Chung et al., 2017; Orth et al., 2018). Entering the first grade is a crucial life transition. The transition from early childhood centers to elementary school also indicates the transition from early childhood to middle childhood. First-graders must adjust to new school environments and find their standing as social and academic beings among their peers. Self-esteem measured at first grade may reflect how children have grown up, while predicting how they will adjust in a school setting (Entwisle et al., 2005).

### The Current Study

Few studies have examined the quality and stability of the responsive parenting practices of mothers with infants and the longitudinal links between these practices and children’s self-esteem. Using data presented by the Panel Study on Korean Children, this study identified Korean mothers’ responsive parenting trajectories from birth to age three and examined the associations with children’s self-esteem at first grade. The study proposed two specific hypotheses. First, Korean mothers with infants will be classified into several distinct groups sharing a similar intensity and stability in their responsive parenting. This assumption is based on other related literature classifying normative samples of people into several distinct groups displaying similar intensity and stability in behavioral patterns. Like other human behaviors or traits, responsiveness in maternal parenting is anticipated to change over time. This study intends to capture the within-group changes and the between-group differences in maternal responsive parenting as multiple trajectories. Second, it is expected that first-graders who experienced more stable and intense responsive parenting from their mothers from birth to age three will score higher on the self-esteem measure than their peers. This hypothesis is rooted in the concept that more intense experiences over a longer duration at earlier stages of development result in more significant developmental differences; it is also based on the insight that high-quality parenting contributes to developing children’s neuro-psychological foundations in a positive manner.

## Materials and Methods

### Participants

The analyses for this study are based on data from the Panel Study on Korea Children (PSKC). The PSKC is an ongoing nationwide data collection project conducted by the Korea Institute of Child Care and Education. The PSKC provides comprehensive information on child development, parenting, family function, and policy effectiveness in Korean households. A total of 2,150 households with infants born between April and July 2008 were sampled using a stratified multi-stage method. The sample retention rate when children reached age 7 was 74.3%. The characteristics of participants are presented in Table 1.

**Table 1:** Descriptive characteristics of participants.

Characteristics	Maternal responsive parenting trajectory group						Total		Test statistics
	Low		Moderate		High				
	*M* (*SD*)	%	*M* (*SD*)	%	*M* (*SD*)	%	*M* (*SD*)	%
Child sex (boy)^a^		53.8		50.2		49.0		50.7	*X*^2^_(2, *N* = 2,101)_ = 2.058
Birth weight (kg)^a^	3.265 (0.422)		3.255 (0.401)		3.274 (0.435)		3.260 (0.410)		*F*_(2, 2021)_ = 0.307
Birth complication (yes)^a^		11.5		14.0		15.9		13.8	*X*^2^_(2, *N* = 2018)_ = 2.943
Maternal education (2 year college and more)^b^		58.2		75.0		69.9		70.9	*X*^2^_(2, *N* = 1,584)_ = 33.411^***^
Paternal education (2 year college and more)^b^		65.2		75.4		75.2		73.3	*X*^2^_(2, *N* = 1,559)_ = 13.155^**^
Maternal employment (yes)^b^		48.7		43.4		43.9		44.6	*X*^2^_(2, *N* = 1,562)_ = 2.781
Family monthly income (Korean won)^b^	431.643 (210.633)		466.393 (197.545)		489.953 (200.852)		462.566 (201.348)		*F*_(2, 1,570)_ = 5.976^***^
Maternal depressive symptoms^b^	12.455 (4.762)		10.909 (4.223)		9.724 (4.032)		11.057 (4.384)		*F*_(2, 1,547)_ = 27.226^***^
Behavioral problems^b^	19.416 (14.812)		14.170 (12.560)		12.052 (18.486)		14.935 (14.152)		*F*_(2, 1,567)_ = 22.323^***^
Child reported self-esteem^b^	16.595 (2.715)		17.328 (2.348)		17.592 (2.621)		17.215 (2.484)		*F*_(2, 1,552)_ = 13.551^***^

a Child age at 0.

b Child age at 7.

** *p* < 0.01;

*** *p* < 0.001.

### Maternal Responsive Parenting

Maternal responsive parenting was measured with six items excerpted from the Korean version of the Parenting Style Questionnaire (Bornstein, 1989; Bornstein et al., 1996; Korea Institute of Childcare and Education, 2022). Using the six items (e.g., I understand what my child wants or how he/she feels; I promptly and appropriately respond to my child’s expressed distress or discomfort), mothers rated their responsiveness from 1 (hardly at all) to 5 (all the time) at age 0, 1, 2, and 3. High scores indicated that mothers display prompt, affectionate, and appropriate responses when they interact with their children. The internal consistencies (Cronbach’s alpha) of the six items across four years were 0.820, 0.833, 0.847, and 0.830, respectively.

### Self-Esteem of Children

Five items from the Rosenberg self-esteem questionnaire (Rosenberg, 1965; Korea Institute of Childcare and Education, 2022) translated into Korean were employed for this study. The original Rosenberg self-esteem questionnaire consisted of ten items. The PSKC research team selected five items from the ten to measure first-graders’ self-esteem in reference to the prior work of the Millennium Cohort Study in the UK. To measure the self-esteem of first-graders, trained interviewers visited children’s homes and guided them to respond to each question from 1 (strongly disagree) to 4 (strongly agree). The internal consistency (Cronbach’s alpha) of the five-item self-esteem questionnaire for this study was 0.77. Higher scores indicated that the first-graders had more positive self-esteem.

### Analyses

The current study followed the standard three-step method proposed by Van De Schoot et al. (2017). First, mothers’ responsive parenting trajectories from birth to age three were identified with latent class growth analysis by assuming the variances and covariances of the growth factors within each class were zero. By adopting the unconditional latent class growth analysis, the current study explored the number of latent classes without considering covariates. It enables future studies to replicate the research and compare results. Missing values were treated using maximum likelihood estimation under missing completely at random, the default function in Mplus 8.0. Second, the most likely class membership of maternal responsive parenting was saved as the independent variable into the main data. Third, for exploring differences in children’s self-esteem by maternal responsive parenting trajectories, analysis of covariance was conducted separately from the latent trajectory modeling. The most likely class membership of maternal responsive parenting was used as the fixed factor and children’s self-esteem as the dependent variable. Bonferroni multiple comparison tests were used for *post hoc* comparison.

### Covariates

Several variables likely to be linked to mothers’ parenting behaviors or children’s self-esteem were entered into analysis of covariance. Child sex (boys, girls), child birth weight (kg), and birth complications (i.e., intensive care unit treatment or hospitalization: yes, no) were extracted from the first-wave data (child age 0). Maternal and paternal education (2-year college education and more: yes, no), maternal employment (yes, no), family monthly income (Korean won), mothers’ depressive symptoms, and children’s behavior problems were extracted from the sixth-wave data (child age 7).

Mothers’ depressive symptoms were measured with the Korean version of the Kessler Screening Scale for Psychological Distress (K-K6; Kessler et al., 2003; Korea Institute of Childcare and Education, 2022), which is a six-item self-reporting instrument. Mothers rated their emotional states over the past 30 days with 5-point Likert scales. The internal consistency (Cronbach’s alpha) was 0.919. Children’s behavioral problems were measured with the Korean version of the Child Behavior Checklist for Ages 6–18 (CBCL 6–18; Achenbach and Rescorla, 2001; Oh et al., 2010). The Korean version of CBCL 6–18 consisted of 120 items, and mothers rated their children’s behavior from 0 (not true) to 2 (very true or often true). The total behavior problem scores from the Korean version of CBCL 6–18 were utilized for the present study.

## Results

### Trajectories of Maternal Responsive Parenting From Birth to Age Three

As a process for identifying the best group model for maternal responsive parenting trajectories, one- to five-group models were consecutively tested both in linear and quadratic shapes with latent class growth analysis. Statistical indices including entropy (close to 1), Bayesian information criteria (BIC; the smaller the better), the smallest class size (at least 5% of the total cases), bootstrap likelihood ratio tests (BLRT), and Lo–Mendell–Rubin likelihood ratio tests (LMR-LRT) were considered for determining the best group model (Jung and Wickrama, 2008). The overall rationality for the classification and independence between trajectories in each model was graphically judged using plots of sample and estimated means.

**Figure 1:**
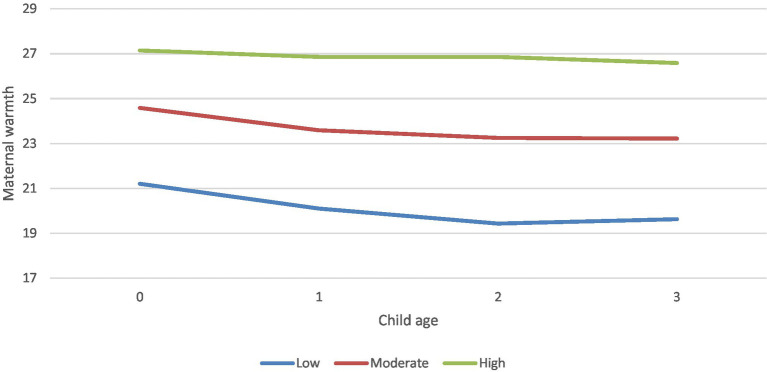
Trajectories of maternal responsive parenting from birth to age 3: sample means.

Statistical indices and graphics of the latent class growth analyses indicated that the three-class quadratic model best describes Korean mothers’ responsive parenting from birth to age three (Table 2). The four-class models showed the highest entropy and the lowest BIC; however, they found classes with less than 5% of the total cases. The three-class models displayed acceptable BLRT and LMR-LRT results and found no classes with less than 5% of the total cases. The entropy and BIC of the three-class quadratic model were better than those of the three-class linear model. The plots of sample means graphically indicate independence between trajectories (Figure 1).

**Table 2:** Statistical indices for 1–4 trajectories of maternal responsive parenting from child age 0 to 3.

Number of trajectories		BIC	Entropy	The smallest class (% of total cases)	BLRT *p* value	LMR-LRT *p* value
1	Linear	36530.221				
	Quadratic	36530.221				
2	Linear	35217.756	0.683	30.319	0.0000	0.0000
	Quadratic	35171.398	0.684	30.462	0.0000	0.0000
3	Linear	34695.771	0.734	14.755	0.0000	0.0000
	Quadratic	34638.384	0.739	14.755	0.0000	0.0000
4	Linear	34591.506	0.770	1.142	0.0000	0.0000
	Quadratic	34531.276	0.775	1.190	0000	0000

In the three-class quadratic model (Table 3), the majority of mothers (66.0%) displayed a moderate level of responsiveness in their parenting. The sample mean of their responsive parenting scores at child age 0 was 24.584, and 23.218 at age three. The differences in responsive parenting scores within the trajectory are significant. The responsive parenting scores decrease from birth to age three with meaningful linear and quadradic changes (Slope = −1.168, *p* < 0.001, Quadradic = 0.242, *p* < 0.001). This group of mothers is considered to have *moderate responsive parenting* trajectory. About 19% of mothers displayed a low level of responsive parenting. The sample mean of their responsive parenting scores at child age 0 was 21.203, and 19.626 at age three. There were significant differences in the responsive parenting scores within the trajectory. Their responsive parenting scores decreased from birth to age three with meaningful linear and quadradic changes (Slope = −1.512, p < 0.001, Quadradic = 0.323, p < 0.001). This group of mothers is considered to have *low responsive parenting* trajectory.

**Table 3:** Sample means of maternal responsive parenting from child age 0 to 3 by trajectories (*n* = 2,101).

Child age	Low *N* = 405, 19.3%	Moderate *N* = 1,386, 66.0%	High *N* = 310, 14.8%
0	21.203	24.584	27.142
1	20.101	23.588	26.858
2	19.432	23.248	26.854
3	19.626	23.218	26.584
Intercept (*SE*)	21.230 (0.257)^***^	24.563 (0.112)^***^	27.110 (0.147)^***^
Slope (*SE*)	−1.512 (0.257)^***^	−1.168 (0.105)^***^	0.179 (0.271)
Quadradic (*SE*)	0.323 (0.073)^***^	0.242 (0.033)^***^	0.004 (0.080)

*** *p* < 0.001.

Approximately 15% of mothers displayed a constant and high level of responsive parenting. The sample mean of their responsive parenting scores at child age 0 was 27.142, and 26.584 at age three. Unlike in the other trajectories, no significant differences in responsive parenting scores existed within the trajectory (Slope = −0.179, Quadradic = 0.004). This group of mothers is considered to have high *responsive parenting trajectory.*

### Self-Esteem of First-Graders by Maternal Responsive Parenting Trajectories From Birth to Age Three

Simple correlation results between maternal responsive parenting scores and child self-esteem at first grade are presented in Table 4. The correlations between maternal responsive parenting scores across four years are significant. Also, first graders’ self-esteem was positively correlated with maternal responsiveness scores. As Table 5 presents, the main effects of the maternal responsive parenting trajectories are statistically valid in first graders’ self-esteem, after controlling confounders. The *post hoc* comparison results (Table 6) revealed that first graders whose mothers have the low responsive parenting trajectory display significantly lower self-esteem than children whose mothers have the moderate responsive parenting trajectory or the high responsive parenting trajectory. However, there is no statistically meaningful difference in self-esteem between children whose mothers have the moderate responsive parenting trajectory and children whose mothers have the high responsive parenting trajectory.

**Table 4:** Correlations between maternal responsive parenting scores from birth to age 3 and child self-esteem at first grade (*n* = 1,560).

S. No.	Variables	1	2	3	4	5
1.	Maternal responsive parenting score at child age 0	1	0.515^***^	0.446^***^	0.434^***^	0.091^**^
2.	Maternal responsive parenting score at child age 1		1	0.544^***^	0.505^***^	0.062^*^
3.	Maternal responsive parenting score at child age 2			1	0.590^***^	0.080^**^
4.	Maternal responsive parenting score at child age 3				1	0.117^***^
5.	Child self-esteem at first grade					1

* *p* < 0.05;

** *p* < 0.01;

*** *p* < 0.001.

**Table 5:** Main effects of maternal responsive parenting trajectories on self-esteem of first graders.

Variables	Self-esteem (*n* = 1,403)	
	Partial *η*^2^	*p* Values
Child sex	0.014	0.000
Birth weight	0.001	0.286
Birth complication	0.001	0.309
Maternal education	0.000	0.964
Paternal education	0.002	0.144
Maternal employment	0.001	0.371
Family monthly income	0.002	0.065
Maternal depressive symptoms	0.004	0.026
Behavioral problems	0.003	0.028
Trajectories of maternal responsiveness parenting	0.014	0.000
	*F*_(2,1,391)_ = 10.152^***^	

*** *p* < 0.001.

**Table 6:** Self-esteem of first graders by trajectories of maternal responsive parenting (*n* = 1,403).

	Trajectory group			Group comparison
	Low (a)	Moderate (b)	High (c)	
	*M* (*SE*)	*M* (*SE*)	*M* (*SE*)	
Self-esteem	16.691 (0.144)	17.344 (0.078)	17.619 (0.174)	a < b^***^ a < c^***^

Adjusted variables are child sex, child birth weight, child birth complication, maternal education, paternal education, maternal employment, family monthly income, maternal depressive symptoms, and behavioral problems of first graders.

*** *p* < 0.001.

A series of analyses of covariance were additionally conducted to compare the effect size of the maternal responsive parenting trajectories with those of the maternal responsive parenting scores each year. As presented in Table 7, the effect size of the maternal responsive parenting trajectories was larger than those of maternal responsive parenting scores at childbirth, age 1, age 2, and age 3.

**Table 7:** Effect sizes (Partial *η*^2^): trajectories of maternal responsive parenting vs. maternal responsive parenting scores.

Maternal responsive parenting trajectories	vs.	Maternal responsive parenting score at child age 0
0.011^***^		000
Maternal responsive parenting trajectories	vs.	Maternal responsive parenting score at child age 1
0.016^***^		0.002
Maternal responsive parenting trajectories	vs.	Maternal responsive parenting score at child age 2
0.013^***^		0.001
Maternal responsive parenting trajectories	vs.	Maternal responsive parenting score at child age 3
0.005^*^		0.001

No adjusted variables.

* *p* < 0.05;

*** *p* < 0.001.

## Discussion

This study was conducted with two specific goals. The first goal was to identify responsive parenting trajectories among Korean mothers from birth to age three. The second goal was to examine the associations between mothers’ responsive parenting trajectories and children’s self-esteem at first grade. Here the researcher presents those findings and implications. A discussion of the present study’s limitations and suggestions for intervention practices and future research will follow.

First, as expected, distinct multiple responsive parenting patterns appeared among Korean mothers with infants. Korean mothers developed one of three responsive parenting trajectories from birth to age three: low (19%), moderate (66.0%), or high (15%). The current findings propose that mothers’ parenting behaviors may be better explained with multiple latent groups rather than a single path model. Regarding the stability of responsiveness, in this study, mothers practicing the highest level of responsiveness at childbirth maintained that quality of parenting up to toddlerhood. On the other hand, mothers practicing the moderate or the low level of responsiveness at childbirth did not maintain or increase the intensity of their responsiveness. Previous studies on stability in parenting quality have yielded inconsistent results. For example, Roskam and Meunier (2012) reported that the supportive parenting of mothers with children aged two to nine years decreased over time. Ettinger et al. (2018), on the contrary, claimed that the parenting practices of low-income ethnically diverse mothers with children under age five improved over time as the children aged. Cook et al. (2012) claimed that the parenting practices of Early Head Start mothers were stable across three time points (at 14, 25, and 36 months). The discrepancies between studies are probably due to differences in their sample populations’ characteristics, children’s ages, parenting behavior measures, numbers of trials of the data collection, and time intervals between the data collection (King et al., 2018). The current findings suggest that multiple longitudinal responsive parenting patterns exist in the normative sample of mothers with infants, and that each group is distinct in its stability of responsiveness.

It is notable that the hierarchy of responsiveness of the three groups remained stable from birth to age three. No group of mothers showed a dramatic increase or decrease of responsive parenting practices, though there might have been some meaningful mean-level changes within the middle and the low trajectories. The maintenance of the hierarchy between trajectories has been observed in studies of personality developmental paths (Specht et al., 2011). On the other hand, in studies addressing clinical issues such as depressive symptoms (Kim, 2017) or alcohol use (De Genna et al., 2017), groups of people often display dramatic changes in symptoms or behaviors and cross the trajectories of the other groups. The current findings suggest that between-trajectory differences in maternal responsive parenting may be minimal in a normative sample, yet the within-trajectory differences may differ by the initial level of responsiveness.

These features of maternal responsive parenting impose both challenges and opportunities for parent intervention fields. The goal of parent interventions is to promote child outcomes by adjusting parenting behaviors. For some mothers, such as mothers with low responsiveness in parenting at childbirth, stabilizing their parenting responsiveness to the level practiced at childbirth may be difficult. Intervention practitioners must have a thorough understanding of the parenting features of their target group and should set appropriate goals and matching intervention strategies. On the other hand, it is promising that when high responsiveness in parenting is established at childbirth, mothers tend to maintain that quality during early childhood. Thus, for at-risk parents, strong alliances with practitioners need to be forged as early as possible (Landry et al., 2008). During pregnancy or early infancy are considered the best times for responsive parenting practice interventions.

Second, there was a significant main effect of maternal responsive parenting trajectories from childbirth to age three in children’s self-esteem at first grade. The main effect was valid after controlling several meaningful confounders such as child sex, maternal depression, and children’s behavioral problems. First-graders who experienced the low level of maternal responsive parenting from birth to age three had significantly lower explicit self-esteem as compared to first-graders who experienced the moderate or the high level of maternal responsive parenting. The current findings confirm the existing claim that parenting is associated with children’s self-esteem (Milevsky et al., 2007; Pinquart and Gerke, 2019). So far, studies reporting meaningful links between parenting and the self-esteem of children have mostly employed cross-sectional research designs, which leaves uncertainty in the casual direction. The current findings underscore that experiencing stable and responsive parenting from mothers during infancy contributes to later positive and healthy self-esteem development. Also, children’s self-esteem in first grade was better explained by the longitudinal trajectories of maternal parenting practices than those observed in a single time point. This highlights the value of examining latent trajectories of parenting practices in predicting children’s outcomes.

Meanwhile, there are no meaningful differences in self-esteem between the children of mothers with the moderate responsive parenting trajectory (the majority group of children) and children of mothers with the high responsive parenting trajectory. The level of responsiveness and the stability observed in the high trajectory group of mothers is desirable, but these may not be the goals or standards applicable to all parents. For children who struggle with developing positive judgments about their self-worth in attachment relations in family contexts, active compensative interventions may be necessary. Considering self-esteem in early adolescence predicts mental health outcomes in late adolescence and early adulthood (Masselink et al., 2018); low self-esteem may indicate unstable developmental foundations or act as a forerunner of other psychological issues. Developmental plasticity is still high in middle childhood (Buttelmann and Karbach, 2017), and self-esteem changes across one’s lifespan. Safe and encouraging outside-home relationships may be beneficial for these children. In middle childhood, social bonds with outside-home family members have special meaning in children’s lives. Peers and adults can play a crucial role as attachment figures. In close, secure, and reliable relationships with peers and adults, children can build or reshape their self-concepts as worthy and acceptable (Chu et al., 2010; Gorrese and Ruggieri, 2013).

Another emerging intervention approach is cognitive training. A recent study reported that a three-month socio-cognitive training intervention for increasing meta-cognitive perspectives on the self and others induced changes in participants’ emotional self-descriptions and concomitant structural changes in the brain regions related to self-concept (Lumma et al., 2018). Training or programs promoting direct changes in children’s brain structures related to self-esteem can be considered as options for self-esteem interventions, though intensive empirical research should be conducted to prove their validity and long-term effectiveness for children (Rossignoli-Palomeque et al., 2018).

Taken together, this study identified three longitudinal responsive parenting patterns in a normative sample of Korean mothers with infants: high, moderate, and low. Responsiveness in maternal parenting behavior has longitudinal rank-order stability, indicating that the between trajectory differences are minimal. The within-trajectory differences vary according to the initial levels of maternal responsiveness. The group of mothers showing the highest responsiveness at the time of childbirth maintained the intensity of their responsiveness as children aged; the other groups of mothers did not maintain or increase their responsiveness. Parental intervention practitioners should understand the unique features of the maternal parenting practices of their target groups and set appropriate goals and strategies. Meaningful links were found between maternal responsive parenting trajectories from birth to age three and children’s self-esteem at first grade. Children of mothers with the low responsive parenting trajectory reported significantly lower self-esteem at first grade than their peers. Low self-esteem in middle childhood linked to prior low-quality maternal parenting may indicate a vulnerability in neuro-psychological foundations. These children should be prioritized in compensatory out-home interventions for building up sound self-concepts and enhancing developmental foundations.

The findings of this study should be considered in light of several limitations. First, the attrition of data may affect the results of this study. The analysis for identifying maternal responsive parenting trajectories utilized 2,101 cases, but the analysis for the main effects of the trajectories on first-graders’ self-esteem employed 1,403 cases. According to an attrition analysis about the PSCK (Lim et al., 2022), high-income and well-educated families were more likely to continue participating in the PSKC. The current findings may better represent functioning children and mothers, which lessens the generalizability of the results. Second, maternal responsive parenting was measured using the six-item self-report instrument proposed by the PSKC research team. The self-report method is susceptible to respondents’ subjective biases and social desirability. Also, the six items can hardly reflect the complex constructs of responsive parenting. Similarly, the self-esteem of children was measured with five items extracted from the original Rosenberg self-esteem scale, which limits comparisons with the previous research using the original scale. Third, most confounders (i.e., family monthly income, parental education, maternal employment, maternal depressive symptoms, and behavioral problems of children) were extracted when the children were 7 years old. The role of confounders from childbirth may not be fully reflected in the longitudinal relationship between maternal responsive parenting and the self-esteem of children. Fourth, though several confounders were controlled, other meaningful variables might modify the longitudinal paths of maternal responsive parenting or affect children’s self-esteem. For example, paternal parenting involvement or parental support may affect the quality or stability of maternal parenting. Stimuli and responses from fathers during infancy may influence children’s neuro-psychological foundational development, resulting in self-esteem differences in later years. Children’s individual characteristics, such as temperament and physical health conditions, might have a transactional impact on maternal parenting practices and impact the development of their own self-esteem. Further studies should incorporate a diverse range of variables to portray a comprehensive picture regarding parenting and children’s self-esteem development.

This study is one of few to identify maternal responsive parenting trajectories in a nationally representative sample. The varied intensity and stability of responsiveness captured in the three parenting trajectories produce meaningful echoes regarding the nature of parenting behaviors and how to help mothers with poor parenting responsiveness. Differences in early maternal responsive parenting in terms of stability and intensity are associated with differences in the self-esteem of school-aged children. Further works should clarify the biological, environmental, and transactional factors that facilitate or weaken the association in a comprehensive frame.

## Data Availability Statement

Publicly available datasets were analyzed in this study. This data can be found at: The Panel Study on Korean Children https://panel.kicce.re.kr/pskc/intro_pskc.do.

## Author Contributions

The author confirms being the sole contributor of this work and has approved it for publication.

## Conflict of Interest

The author declares that the research was conducted in the absence of any commercial or financial relationships that could be construed as a potential conflict of interest.

## Publisher’s Note

All claims expressed in this article are solely those of the authors and do not necessarily represent those of their affiliated organizations, or those of the publisher, the editors and the reviewers. Any product that may be evaluated in this article, or claim that may be made by its manufacturer, is not guaranteed or endorsed by the publisher.
